# Emergency Medicine Intern Education for Best Practices in Opioid Prescribing

**DOI:** 10.5811/westjem.2020.9.48808

**Published:** 2020-12-16

**Authors:** Rebecca Lowy, Ryan P. Bodkin, Rachel Schult, Molly McCann, Courtney Marie Cora Jones, Nicole M. Acquisto

**Affiliations:** * University of Rochester Medical Center, Department of Emergency Medicine, Rochester, New York; † University of Rochester Medical Center, Department of Pharmacy, Rochester, New York; ‡ University of Rochester Medical Center, Department of Public Health, Rochester, New York; § University of Rochester Medical Center, Department of Orthopedics, Rochester, New York

## Abstract

**Introduction:**

Opioid exposure has been identified as a contributing factor to the opioid epidemic. Reducing patient exposure, by altering heavy opioid prescribing patterns but appropriately addressing patient pain, may represent one approach to combat this public health issue. Our goal was to create and implement an opioid education program for emergency medicine (EM) interns as a means of establishing foundational best practices for safer and more thoughtful prescribing.

**Methods:**

This was a retrospective study at an academic, urban emergency department (ED) comparing ED and discharge opioid prescribing practices over a 12-week time period for two 14-intern EM classes (2016 and 2018) to evaluate an early opioid reduction education program. The education program included opioid prescribing guidelines for common ED disease states associated with moderate pain, clinician talking points, and electronic education modules, and was completed by EM interns in July/August 2018. Opioid prescription rates per shift were calculated and opioid prescribing best practices described. We used chi-squared analysis for comparisons between the 2016 and 2018 classes.

**Results:**

Overall, ED and discharge opioid orders prescribed by EM interns were fewer in the 2018 class that received education compared with the 2016 class. ED opioid orders were reduced by 64% (800 vs 291 orders, rate per shift 1.8 vs 0.7 orders) and opioid discharge prescriptions by 75% (279 vs 70 prescriptions, rate per shift 0.7 vs 0.2 prescriptions). The rate of prescribing combination opioid products compared to opioids alone was decreased for ED orders (32% vs 16%, P < 0.01) and discharge prescriptions (91% vs 74%, P < 0.01) between the groups. Also, the median tablets per discharge prescription (14.5 vs 10) and total tablets prescribed (4305 vs 749) were reduced, P < 0.01. There were no differences in selection of opioid product or total morphine milligram equivalents prescribed when an opioid was used.

**Conclusion:**

An opioid reduction education program targeting EM interns was associated with a reduction in opioid prescribing in the ED and at discharge. This may be an effective way to influence early prescribing patterns and best practices of EM interns.

## INTRODUCTION

Use of prescription opioids in the United States (US) has risen sharply over the past 20 years with sales nearly quadrupling from 1999 to 2014.^1^ Similarly, rates of drug overdose deaths increased 140% over the time period 2000–2014 with those resulting from opioid overdose increasing from about three to nine deaths per 100,000 persons.^2^ Over 600,000 people have died as a result of drug overdose since 1996 with about two-thirds involving an opioid.^3^ Understanding the role of overall opioid exposure in the population as a driving factor for the epidemic led to recommendations suggesting a focus on safer prescribing practices in the medical community.^2^ Although clinicians are concerned about opioid misuse among patients, many also report lack of training regarding opioid prescribing and optimal pain management.^4^

Pain is one of the most common complaints among patients presenting to US emergency departments (ED) and is estimated to be reported by over 50% of patients.^5^,^6^ The percentage of overall ED visits where any opioid analgesic prescribed increased from 20.8% in 2001 to 31% in 2010.^7^ The development of chronic pain after an episode of acute pain is concerning as approximately 16% of patients receiving more than a one-week supply of opioids and 6% of patients receiving even a one-day supply reported continued use after one year.^8^ Previous investigations have found a reduction in ED opioid use and prescribing after the implementation of formal programs aimed at this goal.^9^–^11^ Recognizing the potential role of ED use/prescribing in the opioid epidemic and the impact that a formal program can provide, we assembled a pharmacist-led, interdisciplinary task force to create and implement an opioid reduction program with the goal of reducing ED opioid orders and discharge prescriptions by 30%. Using the tools from this program, we sought to evaluate the impact of early education incorporated into emergency medicine (EM) resident training on multimodal pain management and smart opioid use.

## METHODS

Our institution is an 886-bed academic, urban, tertiary care center with a 120-bed ED serving over 115,000 patients annually. An ED-focused opioid reduction program was developed and implemented in November 2017.^12^ The program included opioid prescribing guidelines and evidence-based pathways for multimodal, stepwise pain management with opioid rescue for common ED disease states associated with moderate pain (musculoskeletal pain, back pain, renal colic, fractures, and headaches). The guidelines recommended morphine instead of hydromorphone or oxycodone for less euphoric effects, oral compared to intravenous opioids when possible, use of opioid agents alone compared to combination products (eg, opioid/acetaminophen) for optimization of non-opioid adjuncts and more effective multimodal therapy, and no more than a three-day supply if prescribing opioids at discharge. We created talking points related to pain management and smart opioid use and smart phrases in the electronic health record (EHR) for use in the patient discharge summary. To educate current and new staff on the ED opioid reduction program and available materials, electronic education modules were created. Participants would review materials and presentations and then attest that they were reviewed (Table 1).

The EM medical residency program is a three-year program with 14 residents per class. Early in the 2018 education year (July/August), the EM intern class completed this education. To evaluate the impact of this early education, we conducted a retrospective study comparing ED and discharge opioid-prescribing practices for the 2016 and 2018 intern classes (before and after the ED opioid reduction program/education). Data on opioids prescribed for ED administration or at discharge, including medication name, dose, route of administration, directions, and number of tablets (for discharge prescriptions) was extracted from our EHR for two 12-week time periods, August 12–November 4, 2016 and August 10–November 2, 2018. From this report, we extracted opioids prescribed by EM interns from the 2016 and 2018 classes.

We used intern schedules to identify shifts worked to calculate prescribing rate. A rate of opioids prescribed per shift was calculated for both ED opioid orders and discharge prescriptions. We used a rate since each intern worked a different number of shifts during the time period. Additional analyses on the prescribing of hydromorphone or oxycodone compared to morphine, total morphine milligram equivalents (MME) prescribed, combination products compared to opioids alone, and number of tablets provided at discharge were compared. We used chi-squared analysis to compare the 2016 and 2018 groups for all endpoints. A *P*-value of < 0.05 was considered statistically significant, and all analyses were completed using SAS version 9.4 (SAS Institute, Inc., Cary, NC).

## RESULTS

A total of 28 EM interns were included in the study (14 in each class). Demographic information and shifts worked were similar between the two classes (Table 2). Overall, ED opioid orders were reduced by 64% (800 vs 291 orders, rate per shift 1.8 vs 0.7 orders) and opioid discharge prescriptions by 75% (279 vs 70 prescriptions, rate per shift 0.7 vs 0.2 prescriptions). The rate of prescribing combination products compared with opioids alone was significantly decreased for ED opioid orders (32% vs 16%, *P* < 0.01) and opioid prescriptions at discharge (91% vs 74%, *P* < 0.01) between the 2016 and 2018 groups. Also, the median tablets per discharge prescription were decreased (14.5 vs 10), and total tablets prescribed were reduced by 83% (4305 vs 749) in the study period. There were no differences in hydromorphone/oxycodone prescribing compared to morphine or total MME prescribed when an opioid was ordered.

## DISCUSSION

These data suggest several positive results with education targeting EM interns and influence on opioid prescribing patterns. Specifically, education modules with learning points directly focused on prescribing opioids alone, when needed, compared to a combination product to allow optimization of acetaminophen alone correlated with a reduction in combination product use. We also found substantial reductions in both the number of opioid prescriptions at discharge and median tablets per discharge prescription. This equates to less frequent, shorter courses of opioid analgesics for patients returning to the community and reduces exposure harm that may lead to increased risk of habit formation inherent with more prolonged use.^4^,^8^–^11^ Fewer tablets available in the community may also decrease the incidence of pill diversion. The lack of notable reduction in hydromorphone and oxycodone prescribing compared to morphine or total MME prescribed, if an opioid was ordered, might be explained by the clinical complexity of the patients seen in our ED, pre-existing comfort of clinicians with certain opioids and doses, or saved EHR preferences.

To our knowledge, this is the first report evaluating focused education at the beginning of medical residency training on actual practice changes and the only one to describe EM trainees. Other reports surveyed surgery or ophthalmology trainee opioid and pain management prescribing practices, perceived influences, and knowledge of prescribing resources.^13^–^16^ Additionally, one report evaluated surgical resident prescribing practices via survey immediately before and after a one-hour didactic training focused on opioid prescribing best practices that found trainees answered with more non-opioid pain management options and reduced tablets prescribed when an opioid was selected to be used.^17^

## LIMITATIONS

One potential limitation is that prescribing changes may have occurred in the setting of the larger landscape of the opioid epidemic, which had come much more into the public eye over the study period. Thus, it is possible that incoming interns in the second cohort (2018) were exposed to external factors that may have influenced their perceptions related to ED pain management and the use of opioid analgesics. We do not speculate there were other influences on the opioid prescribing differences seen between the 2016 and 2018 EM intern classes, as the EM intern learning environment was unchanged. Specifically, staffing patterns in the ED, patient population, number of patients seen/hour, and general patient demographic for whom they were providing care were unchanged over the two-year study period. It is possible that the EM attending prescribing practice changes did influence EM intern prescribing. However, the EM attending is not required to approve ED orders or discharge prescriptions written by EM interns for controlled or non-controlled substances. Although there is a discussion regarding the pain management plan, it is the EM interns who determine the agent(s) prescribed, route of administration, dose, directions, duration, and total tablets prescribed.

Other potential limitations include the number of study subjects, as this was limited by EM intern class size; potential regional differences in prescribing patterns as these data are from a single medical center; and the fact that chart abstractors were not blinded to the study hypothesis. Also, the study was not designed to evaluate different educational methods or which methods were most effective. Lastly, the greatest impact on prescribing practices may have occurred immediately following education. It it is unclear whether this effect was sustained over time based on the study period.

## CONCLUSION

Early opioid reduction education to EM interns is associated with significant decreases in initial opioid prescribing practice patterns in the ED and at discharge. The use of opioid/acetaminophen combination products and median number of tablets per opioid discharge prescription were also reduced. An opioid reduction education program could be a low-cost, impactful adjunct to early EM intern training to influence prescribing best practices both in the ED and at discharge.

## Figures and Tables

**Table 1:** Opioid reduction education program for emergency medicine interns.

Electronic Modules	Description
ED opioid guidelines	Restrict opioids to moderate/severe pain Opioid risk assessment tool Opioid adverse effects Smart prescribing recommendations – preference of morphine instead of hydromorphone or oxycodone for less euphoric effects; oral instead of IV administration when possible; combination opioid/acetaminophen products not recommended when opioids are used; guidance for lowest effective dose and short treatment duration when discharge opioids are prescribed (3 days or less)
Multimodal pain management and disease-specific pathways	Emphasis on acetaminophen and ibuprofen/ketorolac around the clock with opioids as needed (if necessary) for acute pain management Pathways for pain management of nephrolithiasis, fractures/joint dislocation, musculoskeletal pain, chronic abdominal pain/gastroparesis, migraine/headache with non-opioid alternatives and opioid rescue
Talking points	Useful phrases to aid in patient and family member discussions related to optimal pain management, risks of opioids, and ED pain plan
Lidocaine IV and ketamine IV presentations	Focus on the rationale for use, available literature, indications, dose and administration, and monitoring

*ED*, emergency department; *IV*, intravenous.

**Table 2:** Emergency medicine intern demographic information.

Demographic Variable	2016 (n = 14)	2018 (n = 14)
Age, mean years ± SD	29.6 ± 4.4	29.0 ± 2.1
Gender, No. male (%)	8 (57)	9 (64)
Degree		
MD, No. (%)	12 (86)	12 (86)
DO, No. (%)	2 (14)	2 (14)
Medical school in the United States, No. (%)	14 (100)	13 (93)
Total ED shifts worked during the study period, No.	447	417

*SD*, standard deviation; *MD*, doctor of medicine; *DO*, doctor of osteopathic medicine; *ED*, emergency department.

## References

[1] Weiss AJ, Wier LM, Stocks C, Agency for Healthcare Research and Quality (US) (2014). Overview of emergency department visits in the United States, 2011: Statistical Brief #174. Healthcare Cost and Utilization Project (HCUP) Statistical Briefs [Internet].

[2] Cordell WH, Keene KK, Giles BK (2002). The high prevalence of pain in emergency medical care. Am J Emerg Med.

[3] Mazer-Amirshahi M, Mullins PM, Rasooly I (2014). Rising opioid prescribing in adult U.S. emergency department visits: 2001–2010. Acad Emerg Med.

[4] Shah A, Hayes CJ, Martin BC (2017). Characteristics of initial prescription episodes and likelihood of long-term opioid use - United States, 2006–2015. MMWR Morb Mortal Wkly Rep.

[5] Weiner SG, Baker O, Poon SJ (2017). The effect of opioid prescribing guidelines on prescriptions by emergency physicians in Ohio. Ann Emerg Med.

[6] Osborn SR, Yu J, Williams B (2017). Changes in provider prescribing patterns after implementation of an emergency department prescription opioid policy. J Emerg Med.

[7] The Colorado Opioid Safety Collaborative (2018). 2017 Colorado Opioid Safety Pilot Results Report.

[8] Shah A, Hayes CJ, Martin BC (2017). Factors influencing long-term opioid use among opioid naïve patients: an examination of initial prescription characteristics and pain etiologies. J Pain.

[9] Brat GA, Agniel D, Beam A (2018). Postsurgical prescriptions for opioid naïve patients and association with overdose and misuse: retrospective cohort study. BMJ.

[10] Manchikanti L, Fellows B, Janata JW (2012). Opioid epidemic in the United States. Pain Physician.

[11] Han B, Compton WM, Blanco C (2017). Prescription opioid use, misuse, and use disorders in U.S. Adults: 2015 national survey on drug use and health. Ann Intern Med.

[12] Acquisto NM, Schult RF, Sarnoski-Roberts S (2019). Effect of pharmacist-led task force to reduce opioid prescribing in the emergency department. Am J Health-Syst Pharm.

[13] Chiu AS, Healy JM, DeWane MP (2018). Trainees as agents of change in the opioid epidemic: optimizing the opioid prescription of surgical residents. J Surg Educ.

[14] Klimczak J, Badhey A, Wong A (2020). Pain management and prescribing practices in otolaryngology residency programs. Am J Otolaryngol.

[15] Olsen KR, Hall DJ, Mira JC (2018). Postoperative surgical trainee opioid prescribing practices (POST OPP): an institutional study. J Surg Res.

[16] Gaspar MP, Pflung EM, Adams AJ (2018). Self-reported postoperative opioid-prescribing practices following commonly performed orthopaedic hand and wrist surgical procedures: a nationwide survey comparing attending surgeons and trainees. J Bone Joint Surg Am.

[17] Yorkgitis BK, Paffett C, Brat GS (2019). Effect of surgery-specific opioid-prescribing education in a safety-net hospital. J Surg Res.

